# Decitabine-based treatment strategy improved the outcome of HSCT in JMML: a retrospective cohort study

**DOI:** 10.3389/fimmu.2024.1426640

**Published:** 2024-08-26

**Authors:** Zhiyong Peng, Jingyu Gao, Litao Huang, Yuelin He, Haoran Tang, Sa Zong, Yanru Pei, Fuyu Pei, Jing Ge, Xuan Liu, Li Yue, Jun Zhou, Xia Li, Dan Yue, Yun Chen, Chen Chen, Xuedong Wu, Xiaoqin Feng, Chunfu Li

**Affiliations:** ^1^ Nanfang-Chunfu Children’s Institute of Hematology & Oncology, TaiXin Hospital, Dongguan, China; ^2^ Department of Pediatrics, Nanfang Hospital, Southern Medical University, Guangzhou, China; ^3^ School of Forensic Medicine, Southern Medical University, Guangzhou, China; ^4^ Pediatric Hematology Laboratory, Division of Hematology/Oncology, Department of Pediatrics, The Seventh Affiliated Hospital of Sun Yat-Sen University, Shenzhen, China; ^5^ Department of Biostatistics, Gobroad Research Center, Shanghai, China

**Keywords:** juvenile myelomonocytic leukemia (JMML), decitabine, hematopoietic stem cell transplantation (HSCT), hypomethylating agents, FLAG protocol, pretransplant therapy, maintenance treatment

## Abstract

**Introduction:**

Pre-HSCT disease control, suboptimal long-term prognosis, and a high recurrence incidence (RI) continue to pose significant challenges for hematopoietic stem cell transplantation (HSCT) in juvenile myelomonocytic leukemia (JMML) patients.

**Methods:**

This retrospective cohort study assessed the effectiveness of a decitabine (DAC)-based protocol in JMML patients undergoing HSCT. The pre-HSCT treatment includes initial and bridging treatment. The efficacy of DAC monotherapy versus DAC combined with cytotoxic chemotherapy(C-DAC) as initial treatment was compared, followed by DAC plus FLAG (fludarabine, cytarabine, and GCSF) as bridging treatment. The HSCT regimens were based on DAC, fludarabine, and busulfan. Post-HSCT, low-dose DAC was used as maintenance therapy. The study endpoints focused on pretransplantation simplified clinical response and post-HSCT survival.

**Results:**

There were 109 patients, including 45 receiving DAC monotherapy and 64 undergoing C-DAC treatment. 106 patients completed bridging treatment. All patients were administered planned HSCT regimens and post-HSCT treatment. The initial treatment resulted in 88.1% of patients achieving clinical remission without a significant difference between the DAC and C-DAC groups (*p=*0.769). Clinical remission rates significantly improved following bridging treatment (*p=*0.019). The 5-year overall survival, leukemia-free survival, and RI were 92.2%, 88.4%, and 8.0%, respectively. A poor clinical response to pre-HSCT treatment emerged as a risk factor for OS (hazard ratio: 9.8, 95% CI: 2.3-41.1, *p=*0.002).

**Conclusion:**

Implementing a DAC-based administration strategy throughout the pre-HSCT period, during HSCT regimens, and in post-HSCT maintenance significantly reduced relapse and improved survival in JMML patients. Both DAC monotherapy and the DAC plus FLAG protocol proved effective as pre-HSCT treatments.

## 1 Introduction

Juvenile myelomonocytic leukemia (JMML) is a rare yet lethal myeloproliferative disease (MPD) of early childhood that is primarily characterized by the overproduction of myelomonocytic cells (1, 2). Allogeneic hematopoietic stem cell transplantation (HSCT) has been firmly established as the main curative approach for most children with JMML (3, 4). Despite this, the long-term survival rates remain suboptimal, with reported overall survival (OS) rates for HSCT being approximately 50-60% and recent reported event-free survival (EFS) 66.4% (5–7). The management of the disease pre-HSCT, particularly the disease control regimen, is contentious and has been a subject of debate. A major cause of treatment failure is the high relapse rate post-HSCT, which exceeds 30% (5). The challenge lies in optimizing both pre-HSCT and post-HSCT regimens to improve overall survival. The pathobiology of JMML is marked by constitutive activation of the Ras signal transduction pathway and dysregulation of genomic DNA methylation (8–10). Given the pathological features of hypermethylation in JMML, hypomethylating agents (HMAs) could potentially enhance therapeutic efficacy (11). This hypothesis was first supported by a report from EWOG-MDS in 2009, where a patient with JMML achieved both clinical and molecular remission after eight treatment cycles with an HMA and remained relapse-free for five years following bridging HSCT (12). The recent prospective AZA-JMML-001 trial demonstrated that azacitidine monotherapy induced a clinical partial response in 11 out of 18 patients and improved leukemia-free survival (LFS) to 82% in 17 patients who underwent HSCT (13). Thus, azacitidine presents a viable option for newly diagnosed children with JMML. The two cytosine analogs, 5-azacytidine (azacitidine, AZA) and 2-deoxy-5-azacytidine (decitabine, DAC), are at the forefront of epigenetic cancer therapies (14). A key difference between these drugs is that DAC is exclusively incorporated into DNA, whereas 80-90% of AZA integrates into RNA (14). Furthermore, a retrospective study indicated improved EFS in patients who received AML-like therapy compared to those receiving no or low-dose chemotherapy (55% vs 32%, *p*=0.048) (6). Additionally, several studies have suggested that post-HSCT maintenance therapy with HMAs is both safe and potentially effective in reducing relapse occurrences in acute myeloid leukemia (AML) (15).

In our study, we assessed the safety and efficacy of a DAC-based treatment strategy in a sizable cohort of 109 children with JMML. This comprehensive treatment approach commenced with a standard dose of DAC as the initial therapy. Subsequently, the treatment incorporated DAC combined with the FLAG protocol as a bridging treatment. For the conditioning regimens of HSCT, DAC-based regimens were employed, followed by low-dose DAC as a maintenance treatment post-HSCT. In addition to evaluating the survival outcomes, the study also focused on the effectiveness of the treatment in controlling the disease pre-HSCT and in reducing the risk of relapse.

## 2 Methods

### 2.1 Study participants and data source

**Figure 1:**
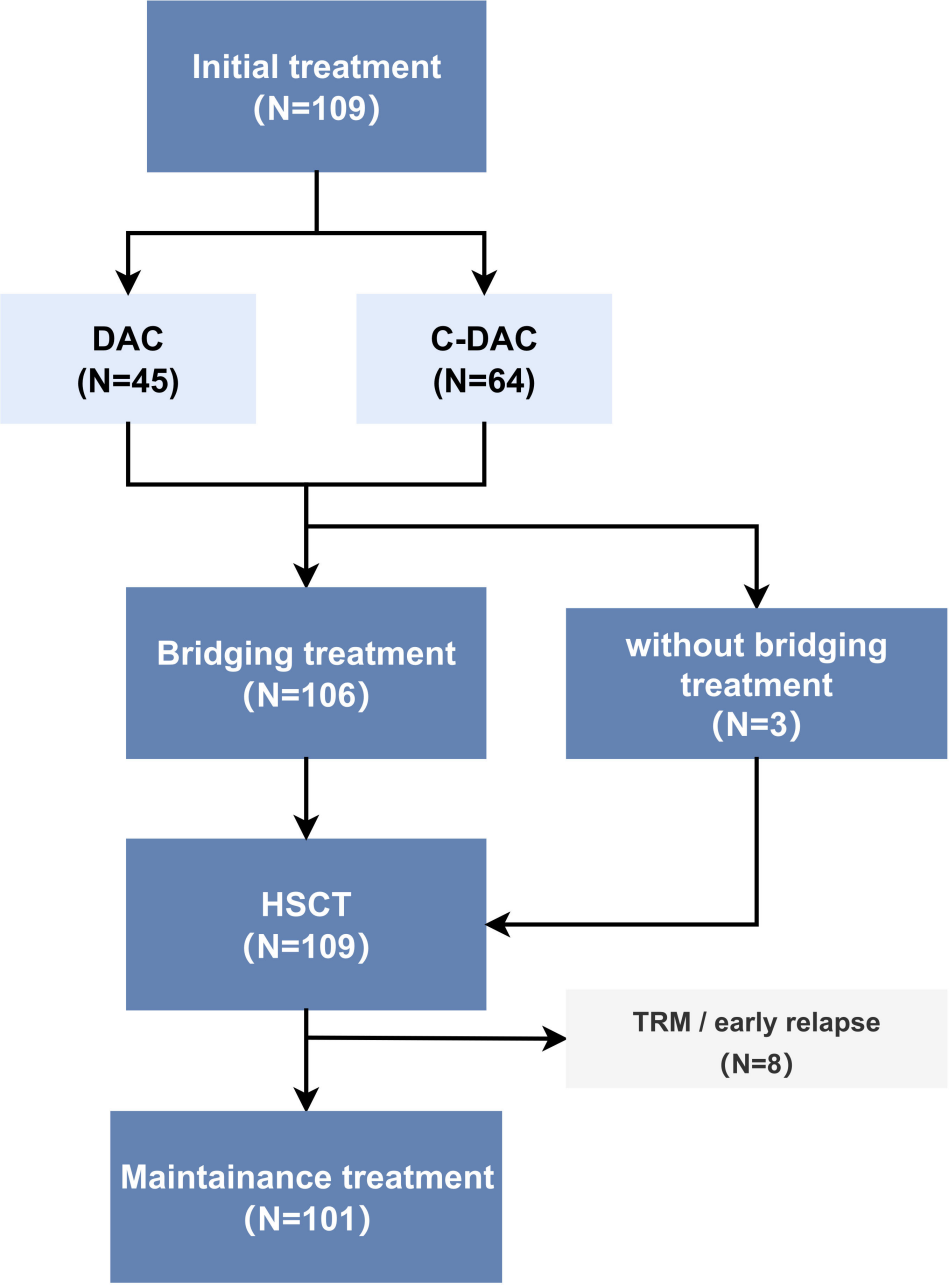
Patient inclusion and study cohort diagram (N=number of patients) TRM (transplant-related mortality), early relapse: relapse within three months after HSCT.

This retrospective, nonrandomized study was conducted in accordance with the principles of the Declaration of Helsinki and received approval from the Medical Ethics Committee of Dongguan Taixin Hospital. Participating centers contributed anonymized data for analysis. We enrolled a total of 109 patients diagnosed with JMML based on the 2016 World Health Organization (WHO) diagnostic criteria (16). These patients underwent HSCT at two different hospitals: Nanfang Hospital, with 31 cases, and Dongguan Taixin Hospital, with 78 cases, between January 2016 and December 2021 (**Figure 1**).

### 2.2 Pre-HSCT treatment: initial treatment and bridging treatment

Two treatment groups were planned and compared during the initial phase of the pre-HSCT treatment: the DAC group (decitabine monotherapy) and the C-DAC group (decitabine combined cytotoxic chemotherapy drugs). DAC: decitabine (20 mg/m^2^/d, 5days), one month per cycle except for severe adverse events. C-DAC: (1) Decitabine (20 mg/m^2^/d, 5days) and cytarabine (200 mg/m^2^/d, 5 days) (partial patients combined with other chemotherapeutic agents, such as homoharringtonine and etoposide). (2) Decitabine and modified A-triple-V regimen (17): decitabine (20 mg/m^2^/d, 5days); modified A-triple-V (Ara-C, etoposide, vincristine) and isotretinoin, one month per cycle, except for severe adverse events. The duration of initial treatment was from the time of diagnosis to the initiation of bridging treatment. Bridging treatment: Patients who were preparing for HSCT received bridging treatment 30-45 days prior to transplantation. Bridging treatment included decitabine and FLAG: decitabine (20 mg/m^2^/d, day 1-5) with FLAG (fludarabine 30 mg/m^2^/d and cytarabine 1 g/m^2^/d day 7-11, granulocyte colony-stimulating factor (G-CSF) 5 μg/kg/d day 6-12). If the WBC count was greater than 25×10^9^/L before bridging treatment, a low dose of cytarabine was initiated to reduce the tumor burden.

### 2.3 HSCT regimens with DAC

The transplantation regimen was divided into three regimens according to the different donors: complementary transplantation (CT), which involved umbilical cord blood (UCB) following haploidentical stem cell transplantation; donor lymphocyte infusion bridging UCB transplantation (LCT), which involved non-G-CSF mobilized haploidentical donor lymphocyte infusion (DLI) bridging unrelated cord blood transplantation; and matched donor transplantation (MDT), which involved peripheral blood stem cell (PBSC) transplantation from matched donors. The criteria to select donors are shown in **Supplementary Table S1**. Conditioning regimens were mainly based on DAC, fludarabine (Flu), and busulfan (Bu) (Bu,4 days or Bu, 3days plus thiotepa, 1 day) (**Supplementary Figure S1**).

### 2.4 DAC as maintenance treatment and NGS as MRD monitoring after HSCT

Maintenance therapy was initiated within 100 days after transplantation when granulocytes were >0.5×10^9^/L and both hemoglobin and platelets remained stable. Decitabine dosage and duration of treatment were adjusted according to neutrophil counts (**Table 1**), 4-6 weeks per cycle, except for severe adverse events. NGS has been used for post-HSCT minimal residual disease (MRD) monitoring and as a reference for therapy adjustment since 2018 (**Supplementary Table S2**).

**Table 1:** Dosage adjustment of post-HSCT Decitabine based on neutrophil counts.

Neutrophil counts	Decitabine Dosage	Duration
≥1.0×10^9^/L	10 mg/m^2^/day	5days
0.5-1.0×10^9^/L or neutropenia (<0.5×10^9^/L) in previous cycle	10 mg/m^2^/day	3days
<0.5×10^9^/L ≥8 weeks*	10 mg/m^2^/day	≤3days

*Neutropenia (<0.5×10^9^/L) persisted for 6-8 weeks: observation.

### 2.5 Study endpoint and definitions

The primary endpoints included leukemia-free survival (LFS), overall survival (OS), primary graft failure (PGF), transplantation-related mortality (TRM), relapse incidence (RI), and graft versus host disease (GVHD). The secondary end point was the proportion of patients with simplified clinical complete remission (scCR) or simplified clinical partial remission (scPR) and variable complete remission (vCR) or variable partial remission (vPR) after pre-HSCT treatment by simplified clinical evaluation (**Supplementary Table S3**), based on international JMML response criteria (18) (**Supplementary Table S4**). Genetic evaluation consisted of cytogenetic response and molecular response based on international JMML response criteria. The cytogenetic response was detected by karyotype analysis. Molecular response was evaluated by three methods: Sanger sequencing (positive/negative), ddPCR (proportion of mutation), and NGS (VAF variant allele frequency, VAF) (**Supplementary Table S5**). Definition of complete remission and relapse after HSCT was based on international JMML response criteria. Primary graft failure was defined as the absence of hematopoietic reconstitution of the donor origin 30 days after HSCT. TRM was defined as the probability of death without relapse, which was the competing event. RI was defined as the probability of relapse. LFS was defined as the time from the date of HSCT until relapse or death, whichever occurred first. OS was defined as the time from the date of HSCT to death or to the cutoff date. Acute GVHD (aGVHD) and chronic GVHD (cGVHD) were diagnosed and graded according to standard criteria (Glucksberg for aGVHD, Original Seattle criteria for cGVHD).

### 2.6 Statistical analysis

The cutoff date for follow-up was 31 December 2022. The data were analyzed using SPSS Statistics software and R (4.1.2). Continuous variables were compared using the t test or the Wilcoxon rank test, and categorical variables were compared with the chi-square test or the Wilcoxon rank sum test in univariate analysis of initial treatment baseline and initial treatment simplified clinical response. The Wilcoxon matched-pairs signed-rank test was applied to compare the difference in clinical response between initial treatment and bridging treatment. The probabilities of TRM were expressed as cumulative incidence (CI), using relapse as a competing event by a competing risk model. Kaplan−Meier curves were used to estimate survival, and the differences in OS and LFS among different groups were compared by the log-rank test. All statistical tests were two-tailed with a significance level of 0.05.

## 3 Results

### 3.1 Patient characteristics

The cohort was divided into two treatment groups: 45 patients in the DAC (decitabine monotherapy) group and 64 in the C-DAC (decitabine combined with chemotherapy drugs) group. The baseline information of two groups is shown in **Supplementary Table S6**. Of these, only 3 patients did not undergo the planned bridging treatment prior to transplantation; two of these three received alternative AML-like chemotherapy instead of the DAC plus FLAG protocol. Excluding those who experienced transplant-related deaths or relapsed within three months post-HSCT, 101 patients underwent post-HSCT maintenance treatment with DAC. The median age of the patients at diagnosis was 23 months, ranging from 1 to 91 months. The study observed a male-to-female ratio of 3.5:1. Patients commonly present with typical JMML clinical symptoms and laboratory features at diagnosis. This included elevated peripheral blood monocyte counts (median, 4.9×10^9^/L) and decreased platelet counts (median, 32.0×10^9^/L). Most patients exhibited typical hepatosplenomegaly and increased levels of hemoglobin F (HbF) (median 23.1%, range 0-78.0%). The most frequently mutated gene among the patients was PTPN11, observed in 57.8% of cases (**Table 2**).

**Table 2:** Characteristics of 109 patients with JMML.

Patient characteristics	
Age at diagnosis, median (IQR), months	23.0(6.5-40.0)
Sex, no. (%) Male Female	85(78.0) 24(22.0)
WBC count, median (IQR), ×10^9^/L monocyte count, median (IQR), ×10^9^/L missing cases, count (%) Hemoglobin, median (IQR), g/L missing cases, count (%) platelet count, median (IQR), ×10^9^/L	33.4(18.2 -54.0) 4.9(3.3-9.7) 14(12.8) 89.0(77.0-102.0) 3(2.8) 32.0(14.0-59.0)
Percentage of HbF, median (IQR), % missing cases, count (%) liver below costal margin, median (IQR), cm missing cases, count (%) spleen below costal margin, median (IQR), cm missing cases, count (%) Lung infiltration(negative/positive), count, % missing cases, count (%)	23.1(6.9- 45.3) 13(11.9) 3.5(2.9-4.6) 4(3.7) 4.0(3.0-6.0) 7(6.4) 30(27.5)/65(59.6) 14(12.8)
Blasts in BM, median (IQR), % Myeloid and erythroid precursors and blast in PB, median (IQR), % missing cases, count (%)	6.2(2.0-10.8) 6.0(2.8-11.0) 27(24.8)
Karyotype, no. (%) Normal -7 +8 others missing cases	67(61.5) 12(11.0) 2(1.8) 6(5.5) 22(20.2)
genetic mutation, no (%) PTPN11 NF-1 KRAS NRAS CBL negative	63(57.8) 20(18.3) 15(11.9) 8(9.2) 1(0.9) 2(1.8)

### 3.2 Efficacy of DAC-based pre-HSCT treatment

During the initial treatment phase, patients receiving DAC monotherapy underwent a median of 3 cycles (ranging from 1 to 10 cycles), while those in the C-DAC treatment group completed a median of 2 cycles (ranging from 1 to 8 cycles). Out of the 109 patients assessed for clinical response after initial treatment, 18 (16.5%) achieved simplified clinical complete remission (scCR), and 78 (71.6%) attained simplified clinical partial remission (scPR), as per simplified clinical evaluation (**Table 3**). The overall response rate, considering both variable complete remission (vCR) and variable partial remission (vPR) for platelets after initial treatment, was 61.4%. Specifically, in the DAC monotherapy group, 8 patients (17.8%) reached scCR, and 31 patients (68.9%) reached scPR. The comparison between the DAC and C-DAC groups showed no statistically significant difference in clinical remission rates (86.7% vs 89.1%, *p=*0.769) (**Table 4**). Furthermore, an exploratory univariate analysis indicated no significant association between baseline characteristics or treatment regimen and treatment responses (**Supplementary Table S7**).

**Table 3:** Clinical response to initial treatment and bridging treatment.

Variables		Initial treatment (N=109)	Paired bridging treatment (N=106)		
			Initial treatment (N=106)	Bridging treatment (N=106)	*P*
WBC		N= 81	N= 80	N= 80	
	vCR	69 (85.2%)	67 (83.8%)	79 (98.8%)	0.083
	vPR	7 (8.6%)	7 (8.8%)	0	
	vSD	5 (6.2%)	6 (7.5%)	1 (1.3%)	
	vPD	0	0	0	
Platelet		N= 96	N= 93	N= 93	
	vCR	39 (40.6%)	38 (40.9%)	59 (63.4%)	<0.001
	vPR	20 (20.8%)	18 (19.4%)	20 (21.5%)	
	vSD	36 (37.5%)	36 (38.7%)	14 (15.1%)	
	vPD	1 (1.0%)	1 (1.1%)	0	
Spleen		N= 98	N= 96	N= 97	
	vCR	16 (16.3%)	16 (16.7%)	28 (28.9%)	<0.001
	vPR	21 (21.4%)	19 (19.8%)	30 (30.9%)	
	vSD	61 (62.2%)	60 (62.5%)	39 (40.2%)	
	vPD	0	1 (1.0%)	0	
Simplified clinical evaluation		N= 109	N= 106	N= 106	
	scCR	18 (16.5%)	18 (17.0%)	21 (19.8%)	0.019
	scPR	78 (71.6%)	75 (70.8%)	82 (77.4%)	
	scSD	12 (11.0%)	12 (11.3%)	2 (1.9%)	
	scPD	1 (0.9%)	1 (0.9%)	1 (0.9%)	

N, numbers of patients enrolled in assessment.

**Table 4:** Efficacy comparison of initial treatment: DAC group vs C-DAC group.

Parameter	Outcome	Total, n (%)	DAC, n (%)	C-DAC, n (%)	*P*
WBC		N=82	N=33	N=49	
	vCR	69(84.1%)	28(84.8%)	41(83.7%)	1.000
	vPR	7(8.5%)	3(9.1%)	4(8.2%)	
	vSD	6(7.3%)	2(6.1%)	4(8.2%)	
	vPD		0	0	
Platelet		N=96	N=38	N=58	
	vCR	39(40.6%)	12(31.6%)	27(46.6%)	0.333
	vPR	20(20.8%)	10(26.3%)	10(17.2%)	
	vSD	36(37.5%)	16(42.1%)	20(34.5%)	
	vPD	1(1.0%)	0	1(1.7%)	
Spleen		N=98	N=38	N=60	
	vCR	16(16.3%)	6(15.8%)	10(16.7%)	1.000
	vPR	21(21.4%)	8(21.1%)	13(21.7%)	
	vSD	60(61.2%)	24(63.2%)	36(60.0%)	
	vPD	1(1.0%)	0	1(1.7%)	
Simplified clinical evaluation		N=109	N=45	N=64	
	scCR	18(16.5%)	8(17.8%)	10(15.6%)	0.870
	scPR	78(71.6%)	31(68.9%)	47(73.4%)	
	scSD	12(11.0%)	6(13.3%)	6(9.4%)	
	scPD	1(0.9%)	0	1(1.6%)	
	scCR/scPR	96(88.1%)	39(86.7%)	57(89.1%)	0.769
	scSD/scPD	13(11.9%)	6(13.3%)	7(10.9%)	

v, variable evaluation; c, clinical evaluation; CR, complete response; PR, partial response; SD, stable disease; PD, progressive disease; n, number of patients.

**Figure 2:**
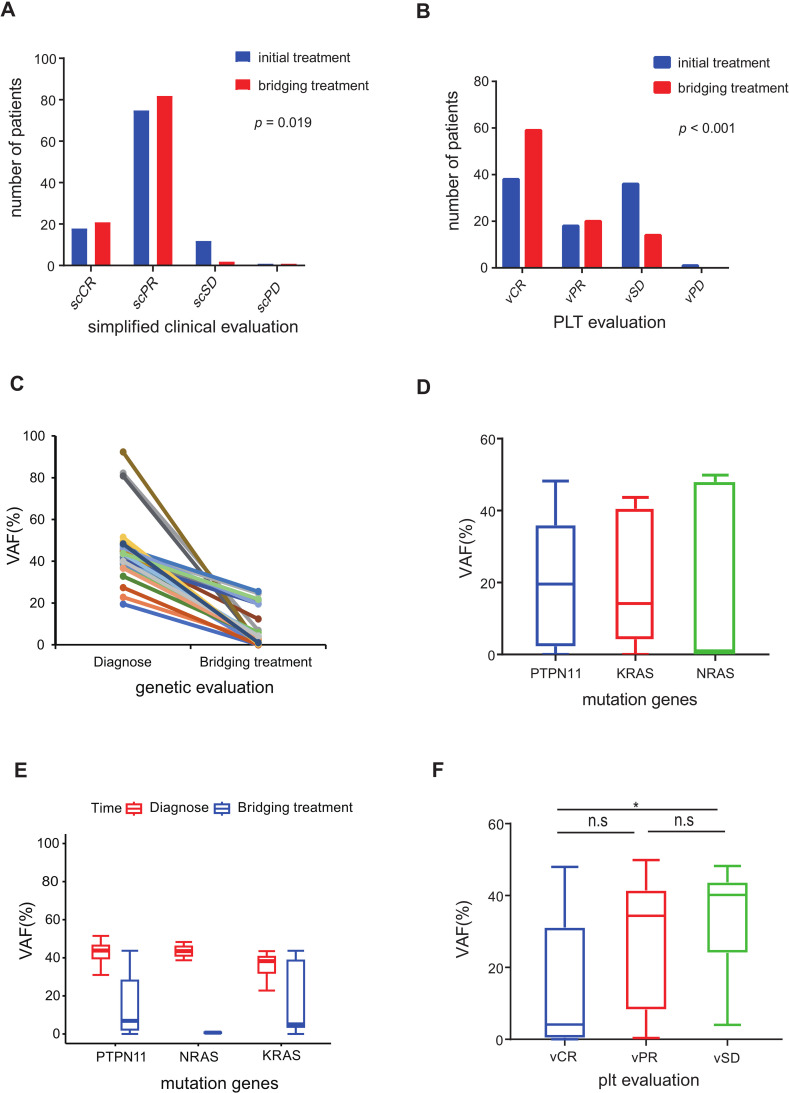
Evaluation of pre-HSCT treatment response **(A)** Simplified clinical evaluation of 106 patients after initial treatment and bridging treatment. **(B)** PLT response evaluation of 106 patients after initial treatment and bridging treatment. **(C)** Diagnosis-bridging treatment match VAF of 31 patients decreased more than 45% after bridging treatment compared to diagnosis. **(D)** The median VAF of the three driver genes (PTPN11, NRAS and KRAS) was under 20% in 64 patients evaluated by NGS after bridging treatment. **(E)** Diagnosis-bridging treatment matched VAF changes in PTPN11, NRAS and KRAS. **(F)** NGS VAF in the different PLT response groups. (n.s *p* >0.05; *p ≤ 0.05).

Out of the 106 patients who underwent bridging treatment, the proportion achieving simplified clinical complete remission (scCR) was 19.8%, and simplified clinical partial remission (scPR) was 77.4%, as shown in **Table 3**. These rates represent an increase of 2.8% for scCR and 6.6% for scPR compared to the initial treatment phase. This improvement suggests that bridging treatment contributed significantly to enhancing the clinical response (*p=*0.019). The positive impact of this treatment was evident in the improved clinical variables, such as white blood cell (WBC) count, platelet count, and spleen size, as demonstrated in **Figures 2A, B** and detailed in **Table 3**.

### 3.3 Genetic evaluation and molecular response

The genetic evaluation in our study encompassed the molecular responses of 80 patients with somatic mutations and the cytogenetic responses of 10 patients (**Supplementary Table S8**). The outcomes of the VAF post-bridging treatment are depicted in **Figures 2C–F**. Among the 43 patients who underwent next-generation sequencing (NGS) analysis with matched diagnosis and bridging treatment data, 31 patients (72.1%) demonstrated a decreasing trend in VAF, with a reduction exceeding 45% from the baseline (**Figure 2C**). In contrast, the VAF in the remaining 12 patients (27.9%) remained relatively stable, showing fluctuations around the original level. A noteworthy observation was the close correlation between platelet response and molecular response (*p* < 0.001). Specifically, patients evaluated with vCR in platelet evaluation exhibited a lower VAF compared to those with vSD (*p* < 0.05, **Figure 2F**).

### 3.4 Primary graft failure, GVHD and survival analysis of HSCT

**Figure 3:**
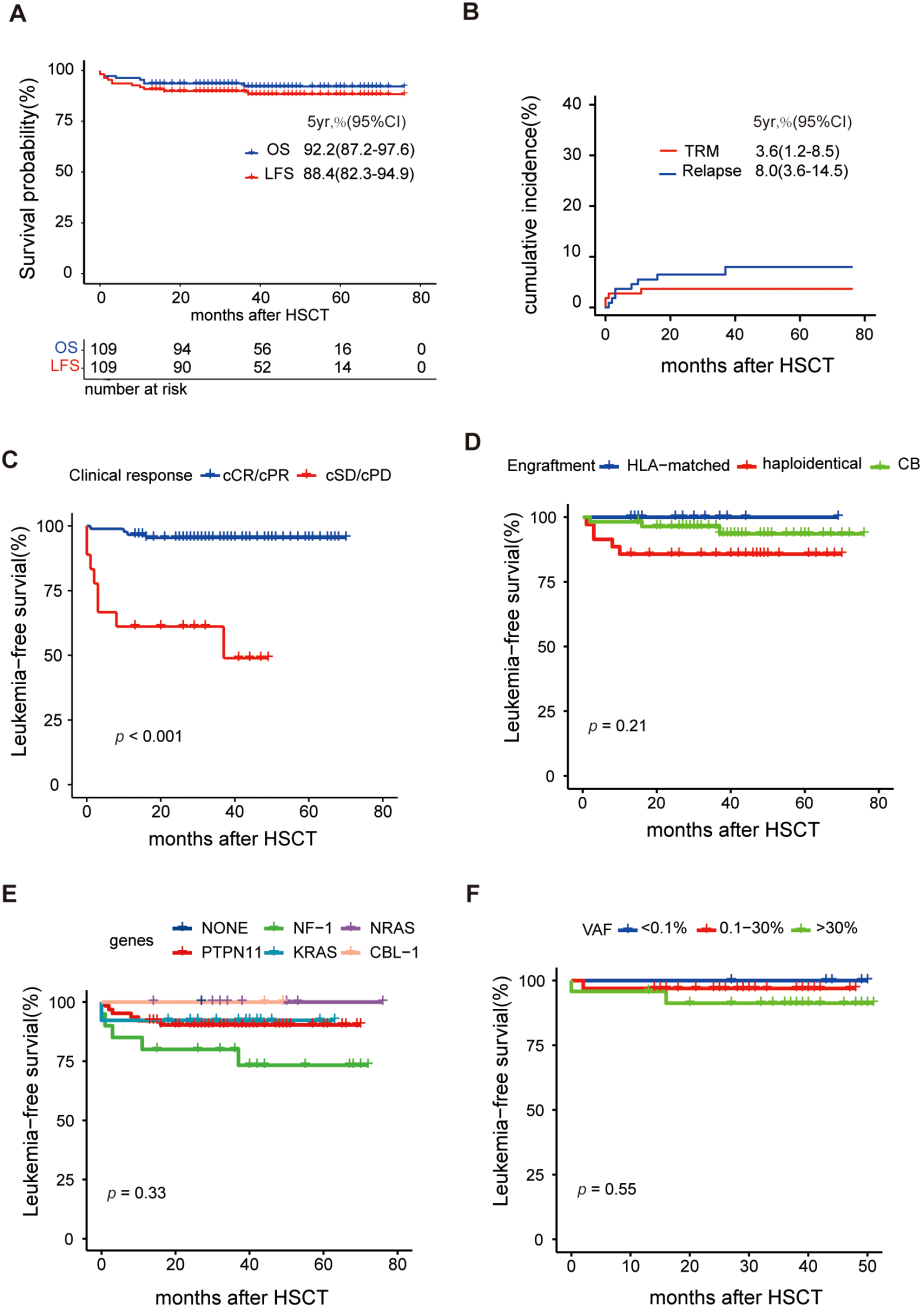
Analysis of survival after HSCT **(A)** Overall survival (OS) and leukemia-free survival (LFS), **(B)** Cumulative incidence of transplantation-related mortality (TRM) and relapse, **(C) **Comparison of LFS between clinical response (scCR/scPR vs scSD/scPD), **(D)** Comparison of LFS between different engraftment, **(E)** Comparison of LFS between different driver genes, and **(F)** Comparison of LFS between different VAFs before HSCT.

**Figure 4:**
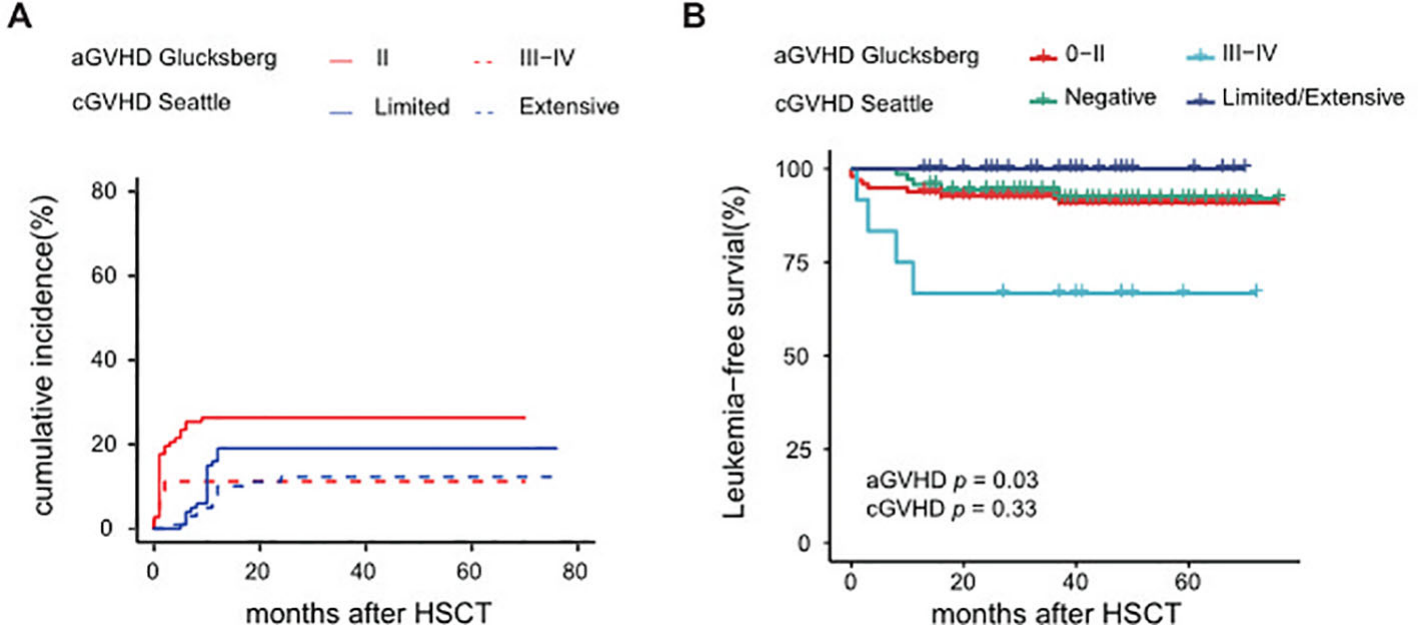
Analysis of GVHD from HSCT **(A)** Cumulative incidence of aGVHD and cGVHD, **(B)** LFS of aGVHD and cGVHD.

Of the 109 patients in this study, the median follow-up was 42 months, ranging from 13 to 76 months. The 5-year OS rate was 92.2% (95% CI 87.0-97.6%), and the 5-year LFS rate was 88.4% (95% CI 82.3-94.9%) (**Figure 3A**). The overall incidence of PGF was 1.9% (95% CI 0.4-6.0%) in this cohort, involving two cases. One of them who did not receive DAC plus FLAG as bridging treatment before HSCT experienced PGF, while another patient failed to engraft umbilical cord blood. After the second transplantation, two patients were successfully engrafted with haploidentical grafts. After successful grafting, the cumulative incidence of TRM was 3.6% (95% CI 1.2-8.5%), and the RI was 8.0% (95% CI 3.6-14.5%), as shown in **Figure 3B**. Eight patients experienced relapse; among them, seven received secondary transplantation, with four achieving leukemia-free survival, while three succumbed to transplantation-related mortality. Additionally, the cumulative incidence of grade II-IV aGVHD was 37.6%, split into 26.3% for grade II and 11.2% for grade III-IV, while cGVHD was observed in 31.4% of cases, comprising 19.2% limited and 12.3% extensive (**Figure 4A**).

Univariate analysis identified several factors related to a higher RI: platelet count <20×10^9^/L, HbF ≥55% at diagnosis, and poor responses to pre-HSCT treatment, as detailed in **Supplementary Table S9**. Furthermore, a poor clinical response to pre-HSCT treatment emerged as a significant risk factor for LFS (**Figure 3C**) and OS with a hazard ratio of 9.8 (95% CI: 2.3-41.1, *p=*0.002). Univariate analysis suggested that grade III-IV aGVHD may lead to poorer LFS, as indicated in **Figure 4B**. Conversely, baseline characteristics, mutant genes, pretransplantation VAF values, HSCT regimens, and donor types did not significantly impact LFS or OS (**Figures 3D–F**, **Supplementary Table S9**). Among 101 patients who were eligible (excluding those with transplant-related death or relapse within three months post-HSCT), the median number of DAC maintenance treatment cycles post-HSCT was six (**Supplementary Figure S2A**). Patients receiving DAC maintenance treatment for ≥9 months exhibited a trend toward a lower RI (0% vs. 7.3%) and improved survival (100% vs. 92.5%) compared to those with less than 9 months of treatment, although the difference was not statistically significant (**Supplementary Figures S2B, C** and **Supplementary Table S9**).

## 4 Discussion

This study represents the first and largest investigation into the use of a DAC-based administration strategy for children with JMML, encompassing the phases of pre-HSCT, conditioning regimens of HSCT, and post-HSCT maintenance treatment. The use of DAC, a hypomethylating agent (HMA), appears to enhance not only pretransplant remission rates but also post-HSCT survival outcomes (12, 13, 19, 20). A cohort of 10 patients reached 71.4% of overall response rate (ORR) after using three cycles of DAC before HSCT (21). Furthermore, a previous prospective trial involving a small cohort of 18 patients demonstrated that AZA induced a 61% cPR prior to HSCT and improved LFS to 82% among 17 patients who underwent HSCT, with a median follow-up of 23.8 months post-HSCT (13). Our retrospective analysis extends these findings, showing that the DAC-based strategy enabled over 80% of our cohort of 109 patients to achieve a clinical response (scCR or scPR) following initial treatment. Moreover, this approach resulted in an OS rate of 92.2% (95% CI 87.2-97.6%), observed over a median follow-up of 42 months post-HSCT.

Previous studies have indicated challenges in managing poor platelet response to chemotherapy and refractoriness to platelet transfusions in JMML (6). In our study, we observed that approximately 60% of patients in both the DAC and C-DAC initial treatment groups showed a significant platelet response, highlighting decitabine’s potential effect on platelet response. Supporting this, research on MDS has shown that decitabine can increase platelet counts by promoting megakaryocyte maturation and enhancing platelet release (22). Moreover, recent findings suggest that decitabine may boost platelet production through immune regulation, particularly by enhancing Treg cell activity and reducing the promoter methylation status of TRAIL, as observed in studies addressing refractory immune thrombocytopenia (23, 24). However, whether HMAs exhibit a similar mechanism in JMML requires further experimental validation.

Previously, clinical studies have reported that cytarabine, either alone or combined with other chemotherapies, could induce leukocyte or splenic remission, with 6-MP and etoposide also showing partial responses (25). Interestingly, in our study, no synergistic effect was observed when combining DAC with cytotoxic chemotherapy during initial treatment. Remarkably, the clinical remission rate reached 87.6% in patients treated solely with DAC monotherapy, suggesting the potential superiority of HMA monotherapy for newly diagnosed patients.

DNA methylation has been linked to high-risk clinical features in JMML, such as older age, high white blood cell count, low platelet count, and mutations in *PTPN11* and RAS-related genes, along with abnormal karyotypes, all correlating with poorer outcomes (20, 26–28). HMAs have been reported to reduce naive leukemic stem/progenitor cells and limit the leukemia-initiating capacity of JMML cells *in vivo* (11). Our study conducted a univariate analysis to assess the influence of clinical baseline factors and driver genes on treatment response, yet no significant differences were identified. This might be due to DAC’s antileukemic and immunomodulatory effects, which seem to improve clinical response and diminish the response disparity among groups, regardless of mutation-related methylation levels.

Engraftment failure has been a persistent challenge in children with JMML undergoing HSCT (29). In the initial phase of our study, a notable case was a patient who received only DAC monotherapy without any additional bridging treatment before HSCT and subsequently suffered from PGF. Interestingly, during the same period, two similar cases of PGF were reported at other medical centers, both in patients who had received only DAC monotherapy before HSCT. This pattern suggests a higher risk of PGF associated with the sole use of DAC monotherapy as a pre-HSCT treatment. Graft failure mediated by residual host T cells may be alleviated by intensive chemotherapy prior to HSCT (30, 31). The role of intensive chemotherapy before transplantation has long been debated. A retrospective COG study highlighted that two patients who underwent aggressive pre-HSCT chemotherapy achieved low disease burden and long-term survival (32). Furthermore, fludarabine and high-dose cytarabine have been reported to provide temporary remission and are well tolerated for leukemia infiltration (3). Consequently, all patients in our study received a combination of DAC and FLAG as bridging treatment prior to transplantation thereafter. After this change, only one patient who underwent UCB transplantation and received bridging treatment experienced PGF, a significant decrease compared to previously reported cohorts (31, 33). The combination of DAC and FLAG regimens as bridging treatment not only improved the clinical response and reduced the tumor burden before HSCT but also facilitated successful engraftment. Therefore, we advocate for a pre-HSCT treatment strategy that incorporates DAC monotherapy as initial therapy and DAC+FLAG as the bridging treatment.

In our approach to JMML treatment, we utilized low doses of DAC as post-HSCT maintenance therapy, aiming to prevent disease recurrence and mitigate GVHD by leveraging the immunomodulatory effects of HMAs. This strategy aligns with findings from a study demonstrating the immunomodulatory properties of low-dose decitabine both *in vitro* and *in vivo* (24). Further research supports the idea that HMAs post-HSCT can reduce GVHD while preserving the graft-versus-leukemia (GVL) effect, primarily by promoting the expansion of regulatory T cells and increasing FOXP3+ Tregs (34, 35). In our cohort, the incidence of extensive cGVHD was 11.1% and did not adversely affect the prognosis.

Although HSCT is considered the primary curative therapy for JMML, relapse remains a major hurdle, often leading to treatment failure (5, 6). Recent studies in AML have begun to show the potential benefits of post-HSCT maintenance therapy (15, 36). Consequently, we adopted DAC-based post-HSCT maintenance therapy as a routine part of our treatment protocol. At a median follow-up of 42 months, the RI in our cohort was observed to be 7.9%, markedly lower than the previously reported rate of 30% (5). Notably, our findings also suggest that patients receiving post-HSCT maintenance therapy for more than 9 months tended to have a lower RI. Reduction of RI can reduce the second HSCT, which is safe but still carries potential treatment risks and increasing expenses (37, 38).

The choice of conditioning regimens in HSCT is crucial for JMML treatment. A randomized trial indicated the superiority of Bu-Cy-Mel over Bu-Flu conditioning regimens, while a retrospective analysis favored Bu/Flu/Mel over TBI-MAC (32, 39). In our study, no significant differences in prognosis were observed among the three HSCT regimens or different types of engraftment. This suggests that the DAC/Flu/Bu conditioning regimen is similarly effective. However, in the CT regimen, patients with haploidentical engraftment appeared to have a higher relapse incidence than those with UCB engraftment (14% vs 6%, *p* = 0.14), as shown in **Supplementary Table S9**. Gerald A.C et al. reported favorable clinical outcomes with the infusion of non-engrafted HLA-haploid CD3+ lymphocytes in the treatment of 41 refractory cases of AML and lymphoma. Possible mechanisms contributing to its effectiveness include initial GVT kill, breaking of host tolerance to tumor through cross-reactive alloreactive responses, persistent nondetectable microchimerism (40). Consequently, we introduced a new LCT regimen to promote UCB engraftment. The reasons for LCT’s lack of superiority may be attributed to the high OS rates in other regimens and a 14% TRM in the UCB engraftment within the LCT cohort, which is comparatively lower than the reported rate of 22% ± 4% (6). Additionally, the smaller size of the LCT cohort, due to its later establishment, may have influenced these results. In JMML, the benefits of UCB, such as lower chronic GVHD incidence and a stronger graft-vs-leukemia effect (41), could outweigh its drawbacks (higher TRM incidence) through optimized management and TRM reduction. Therefore, we believe that the LCT regimen holds future potential in JMML treatment.

Canonical RAS pathway mutations, including *PTPN11*, *NRAS*, *KRAS*, *NF1*, *CBL*, and occasionally *RRAS*, are found in over 95% of JMML patients (42). Next-generation sequencing (NGS) facilitates reliable detection of these mutations, offering comprehensive gene assessment at diagnosis, during complete remission, and at relapse (43). After bridging treatment, NGS-VAF assessments showed a significant decrease, with 9.4% of patients achieving molecular remission. However, pre-HSCT VAF did not impact OS or EFS, indicating that achieving molecular remission before HSCT is not crucial in JMML. Interestingly, molecular response reduction was significantly correlated with platelet response, suggesting that platelet response might be a sensitive indicator of molecular response. Given the low or rare blast cell load at diagnosis, flow cytometry is unsuitable for MRD monitoring in JMML. Since 2018, we have adopted NGS for routine MRD monitoring during posttransplant follow-up to detect early molecular recurrence and adjust treatment accordingly. Further studies are anticipated to validate the clinical value of this approach for JMML in the future.

In summary, our study demonstrates that a DAC-based administration strategy can effectively induce clinical remission and improve overall survival in JMML. DAC as an initial treatment significantly induced high clinical remission rates, particularly in platelets, prior to HSCT. In addition, the combination of DAC with FLAG as a bridging treatment not only further improved clinical remission but also facilitated genetic remission before HSCT. Furthermore, the conditioning regimen incorporating DAC/Flu/Bu proved to be effective for JMML-HSCT. Administering low-dose DAC as post-HSCT maintenance treatment appears to contribute to a reduced relapse incidence. Additionally, tailoring maintenance treatment strategies based on MRD monitoring using NGS could be instrumental in preventing recurrence. The experience gained in managing complications, especially in infants, is also a significant aspect in disease management. Overall, the importance of comprehensive disease management and tailored treatment strategies in JMML cannot be overstated.

Nevertheless, it is important to acknowledge the limitations of this study, which include its median sample size and the retrospective single-arm design. These factors limit the scope of our conclusions, particularly regarding the benefits of post-HSCT maintenance treatment, which necessitates further research. Additionally, given the median sample size and an LFS rate exceeding 88.4%, the incidence of events was too low to conduct a multivariate analysis for identifying risk factors affecting LFS.

## Data Availability

The original contributions presented in the study are included in the article/**Supplementary Material**. Further inquiries can be directed to the corresponding authors.
