# Supplementary material for: Decitabine-based treatment strategy improved the outcome of HSCT in JMML: a retrospective cohort study
Source: Front Immunol. 2024 Aug 26;15:1426640. doi: 10.3389/fimmu.2024.1426640 (PMC11381242; doi:10.3389/fimmu.2024.1426640)
Supplement: Supplementary file 1 [file DataSheet1.pdf]

Supplementary Material

1 Supplementary Data

Supplemental Table 1 Characteristics considered in selecting donors for HSCT

HSCT regimen	Priority of Donor selection	Priority of UCB selection
MDT	**Matched Donor:** -HLA ≥ 9/10	/
CT	**Haploidentical:**<br>-Exclusion of carrying inherited diseases genes<br>-NK cell alloreactive donor (KIR B/x, more B)<br>-Mother (parental donor)<br>-Age (younger donor)	-HLA ≥7/10<br>-NK cell alloreactive donor (KIR B/x)<br>-TNC
LCT	**Haploidentical:** -NK cell alloreactive donor (KIR B/x, more B)	-HLA ≥8/10<br>-NK cell alloreactive donor (KIR B/x)<br>-TNC

MDT: Matched unrelated donor transplantation; CT: Complementary transplantation; LCT: Donor lymphocyte infusion bridging UCB transplantation; UCB: umbilical cord blood; HLA: human leukocyte antigen typing; KIR: Killer cell Immunoglobulin-like Receptors; TNC: Total number of nucleated cells

Supplemental Table 2 Therapy adjustment based on MRD monitoring after HSCT

VAF (NGS) *	Therapy adjustment
0.01-0.1%	reduction or discontinuation of immunosuppression
0.1%-1%	reduction or discontinuation of immunosuppression
	intensification therapy of DAC or DLI
>1%	reduction or discontinuation of immunosuppression
	intensification therapy of DAC and DLI
	prepare for 2nd HSCT and increase NGS evaluation frequency

*Germline mutation (e.g., NF-1) should identify if VAF interference by autologous cell DNA

Supplemental Table 3 Simplified clinical evaluation of non-HSCT treatment in JMML

			Definition of response			Definition of disease progression or relapse applicable to all patients)
		Assessment is feasible if the following are present before therapy	Requirement for CR for each variable (vCR)	Requirement for PR for each variable (vPR)	Requirement for SD for each variable (vSD)	Requirement for PR for each variable (vPD)
Clinical variable	1) WBC count	$>20\times10^9$/L	3.0-15.0$\times10^9$ /L	Decreased by ≥50% over pretreatment but still $>15\times10^9$ /L	not fulfill the criteria of cCR and cPR	Increase by ≥50% and $\geq20\times10^9$ /L
	2) Platelet (Plts) count	$<100\times10^9$/L	$\geq100\times10^9$/L	For patients starting $\geq 20\times10^9$ /L Plts: absolute increase of $\geq 30\times 10^9$/L<br>For patients starting	not fulfill the criteria of cCR and cPR	with Development of transfusion dependency or if patients have the baseline of the Plts count of $\geq30\times10^9$ /L, decrease

				with < 20 $\times 10^9$ /L Plts: increase by ≥ 100% and > 20×10^9 /L		by ≥50% and <100×10^9/L
	3)Spleen size a) Clinical evaluation	≥2cm under the costal margin	No splenomegaly	50% decrease by cm under the costal margin	not fulfill the criteria of cCR and cPR	Increase by ≥100% if baseline <4cm from under the costal margin ≥50% if baseline 5-10cm >30% if baseline >10cm
	b) Sonography	Length of spleen ≥ 150% of upper limit of normal range	Length of spleen ≥ 150% of upper limit of normal range	>25% decrease by length, but still splenomegaly		Increase by ≥25% of length
**Definition of response**						
Simplified clinical complete remission (scCR)	Patient fulfills the criteria of CR of all applicable clinical variables1-3.The response variables must be maintained for at least 2 weeks.					
Simplified clinical partial remission (scPR)	Defined if the patient does not fulfill the criteria of cCR, but vPR was achieved in at least one of clinical variables 1-3 and none of clinical variables showed vPD.					
Simplified clinical stable disease (cSD)	Defined if the patient does not fulfill the criteria of cCR and cPR, but none of the variables showed vPD.					
Simplified clinical progressive disease (scPD)	Defined if any of the variables1-3 showed vPD.					

Supplemental Table 4 International assessment variables of non-HSCT treatment in JMML

			Definition of response		Definition of disease progression or relapse applicable to all patients)
		Assessment of CR and PR is feasible if the following are present before therapy	Requirement for CR for each variable (vCR)	Requirement for PR for each variable (vPR)	Requirement for PR for each variable (vPD)
Clinical variable	1) WBC count	>20×10^9/L	3.0-15.0$\times 10^9$ /L	Decreased by ≥50% over pretreatment but still > 15×10^9 /L	Increase by ≥50% and ≥20×10^9 /L
	2) Platelet count	<100×10^9/L	≥100×10^9/L	For patients starting ≥ 20×10^9/L platelets: absolute increase of ≥ 30×10^9 /L, For patients starting with < 20 $\times 10^9$ /L platelets: increase by ≥ 100% and > 20×10^9 /L	with Development of transfusion dependency or if patients have the baseline of the platelet count of ≥30×10^9 /L, decrease by ≥50% and <100×10^9/L
	3) Myeloid and erythroid precursorsand blastsin PB *	≥5%	0-1%	Decreased by ≥50% over pre-treatment but still ≥ 2%	increase from the baseline: < 5%: ≥ 50% increase and ≥ 5% , ≥ 5%: ≥ 50% increase of total % of myeloid and erythroid precursors and blasts
	4) BM blasts	≥5%	<5%	Decreased by ≥50% over pre-treatment butstill ≥5%	Increase from baseline; < 5%: ≥ 50% increase and ≥ 5% , ≥ 5%: ≥ 50% increase of BM blasts
	5)Spleen size a) Clinical evaluation	≥2cm under the costal margin	No splenomegaly	50% decrease by cm under the costal margin	Increase by ≥100% if baseline <4cm from under the costal margin ≥50% if baseline 5-10cm >30% if baseline >10cm
	b) Sonography	Length of spleen ≥ 150% of upper limit of normal range	Length of spleen ≥ 150% of upper limit of normal range	>25% decrease by length, but still splenomegaly	Increase by ≥25% of length
	6) Extramedullary disease#	Extramedullary leukemic infiltration	No evidence of extramedullary leukemic in any	-	Worsening or new lesions of extramedullary leukemic infiltration

			organ infiltration		
Genetic variables	7) Cytogenetic response	Somaticcytogenetic abnormality detected	Normal karyotype	-	Reappearance or additional acquirement of cytogenetic abnormalities
	8) Molecular response	Somatic geneticanomalies detected	Absence of somatic genetic anomalies acquirement **	-	Reappearance or additional of JMML-specific somatic gene abnormalities
	9) Chimerism response (only for patients after HSCT)	>15% autologous cells after allo-HSCT	Complete donor chimerism		50% increase and >5% increase of autologous cells and >5%

CR: complete response; PR: partial response; PD: progressive disease;WBC:white blood cell; PB: peripheral blood; BM: bone marrow.*Myeloid precursors include promyelocytes,myelocytes and metamyelocytes.The myeloid and erythroid precursors and blasts in PB are given as percentage of the total nucleated cells in PB (WBC including erythroblasts). **In NF-1, PTPN11, NRAS,KRAS,or CBL,thus the mutations are thought to be initiating.In patients with germ-line NF-1,PTPN11 or CBL mutation,only acquired mutations can be evaluated for response and relapse after therapy.The germ-line mutation remains even if patients achieved complete molecular response.#Extramedullary disease includes infiltration of skin,lung,and,very rarely,cranial nerves or central nervous system.

Supplemental Table 5 Methods of gene mutation detection

NGS	DNA from BM was isolated using TIANamp Blood DNA Kit (TIANGEN Biotech (Beijing)Co.,Ltd), amplicon-based sequencing by Ion GeneStudio S5 System (Thermo Fisher Scientific Inc. USA). IGV is used to calculate the variant allele frequencies (VAFs) according to the number of reads of different bases corresponding to the corresponding site, with the human genome hg19 as a reference.
ddPCR	Genomic DNA from BM was isolated using Whole Blood Genome DNA Extraction Kit (Genmagbio Co., Ltd., Beijing, China), and specific mutations were amplified and quantified by DropDx-2044 Digital PCR (Rainsurebio Co., Ltd., Suzhou, China).
Sanger sequencing	Genomic DNA from BM was isolated using Whole Blood Genome DNA Extraction Kit (Feijie Biotechnology Co., Ltd., Shanghai, China), sequenced by DNA AnalyzerABI 3730XL (Thermo Fisher Scientific Inc. USA)

Supplemental Table 6 Baseline of initial treatment regimen

**variables**		**DAC (N=45)**	**C-DAC (N=64)**	***p***
age at diagnosis, months		21.00	23.00	0.861
Sex	Male	34	51	0.644
	Female	11	13	
WBC count, median, $\times 10^9$/L		30.00	37.04	0.077
PLT count, median, $\times 10^9$/L		38.00	29.50	0.200
HbF, median, $\times 10^9$/L		16.15(N=40)	26.20 (N=56)	0.195
Blasts in BM, median, %		6.50	5.50(N=63)	0.680
spleen below costal margin, median, cm		3.80 (N=43)	5.00(N=59)	0.001
Lung infiltration	negative	19	11	0.006
	positive	19	47	
Mutation gene				0.146
	PTPN11	25	38	
	NF-1	10	9	
	KRAS	8	5	
	NRAS	2	9	
	CBL	0	1	
	Negative	0	2	

N=number of patients

Supplemental Table 7 Univariate analysis of clinical response in initial treatment

		cCR/cPR*	cSD/cPD#	*p*
Sex	Male<br>Female	73 (85.9%)<br>18 (75.0%)	12 (14.1%)<br>6(25.0%)	0.221
Age	(mo, median (IQR))	21.00(6.00-39.00)	29.00(9.75-50.50)	0.264
HbF	(%, median (IQR))	21.80(7.90-43.00)	36.30(4.10-57.30)	0.307
WBC	($\times10^9$/L, median (IQR))	34.30(20.04-54.07)	19.75(12.49-47.08)	0.822
Platelet	($\times10^9$/L, median (IQR))	32.00(16.00-60.00)	25.50(12.00-58.75)	0.754
Spleen	(Below costal margin, cm, median (IQR))	4.00(3.00-5.90)	4.0(3.30-9.00)	0.053
Lung infiltration	Negative<br>Positive	25(83.3%)<br>66 (83.5%)	5(16.7%)<br>13(16.5%)	1.000
Driver gene	PTPN11<br>NF1<br>KRAS<br>NRAS<br>CBL<br>NONE	53(84.1%)<br>16(80.0%)<br>11(84.6%)<br>10(100.0%)<br>1(100.0%)<br>0	10(15.9%)<br>4(20.0%)<br>2(15.4%)<br>0<br>0<br>2(100%)	0.078
Karyotype	Normal<br>-7<br>others	75(83.3%)<br>11(91.7%)<br>4(66.7%)	15(16.7%)<br>1(8.3%)<br>2(33.3%)	0.354
Initial treatment regimen	DAC<br>C-DAC	39 (86.7%)<br>52(81.3%)	6 (13.3%)<br>12(18.8%)	0.446

*cCR(clinical complete response)，cPR(clinical partial response)

#cSD(clinical stable disease)，cPD(clinical progressive disease)

p.s: 55 and 43 cases of bone marrow and peripheral blood morphology reached assessment baseline, and only 20 and 16 cases were evaluated after initial treatment

Supplemental Table 8 Genetic evaluation after bridging treatment: Molecular response and Cytogenetic response

**Molecular response**	**PTPN11**	**KRAS**	**NRAS**	**Total(N=80)**
**Sanger seq**	(N=8)	(N=2)	(N=2)	(N=13)
positive	4(50.0%)	2(100.0%)	1(50.0%)	7(53.8%)
negative	4(50.0%)	0	1(50.0%)	6(46.2%)
**ddPCR**	(N=4)	/	/	(N=4)
median (IQR), %	2.8(0.4-2.0)			
**NGS(VAF)**	(N=46)	(N=10)	(N=8)	(N=64)
median ((IQR), %	21.3(3.0-35.4)	25.3(4.7-40.5)	42.4(1.0-48.4)	19.6(2.9-37.5)
<0.1%	3(6.5%)	1(10.0%)	2(33.3%)	6(9.4%)
>0.1%	43(93.5%)	9(90.0%)	6(66.7%)	58(90.6%)
0.1-30%	31(81.5%)	5(13.1%)	2(5.2%)	34(53.1%)
>30%	16(66.6%)	5(20.8%)	3(12.5%)	24(37.5%)
**Cytogenetic response**	-7<br>(N=7)	+8<br>(N=2)	Others<br>(N=2)	Total<br>(N=11)
negative	5	1	2	8
positive	2	1	0	3

* Exclude 17 patient with germline mutation, 2 patients without any typical mutation and 2 patients did not test

Exclude 72 patient without cytogenetic change and 6 patients did not test

N=number of patients

Supplemental Table 9 Transplantation-related mortality (TRM), relapse incidence (RI) and survival analysis

Variable	Value	N	TRM %, (95%CI)	RI %, (95%CI)	5-yr LFS%95%CI	5-yr OS %
		109	3.6(1.2-8.5)	8.0(3.6-14.5)	88.4(82.3-94.9)	92.2(87.2 -97.6)
Age at diagnosis	<25 months	60	5.0(1.3-12.7)	3.4(0.6-10.4)	91.6 (84.9-98.9)	91.1(83.9-98.9)
	≥25 months	49	2.0(0.2-9.5)	13.9(5.3-26.5)	84.1(73.6-96.1)	93.8(87.3-100)
p			0.41	0.07	0.31	0.69
Sex	male	85	3.5(0.9-9.1)	7.1(2.9-14.0)	89.3(83.0-96.2)	92.4(86.7-98.5)
	female	24	4.1(0.3-18.0)	10.7(1.5-30.2)	85.1(70.5-100)	91.7(81.3-100)
p			0.88	0.85	0.80	0.81
WBC count	$<35\times10^9$/L	59	5.1(1.3- 12.9)	10.3(4.1-19.7)	84.6(75.9-94.4)	86.9(78.1-96.8)
	$\geq35\times10^9$/L	50	2.0(0.2-9.3)	4.7(0.8-14.5)	93.3 (86.1-100)	98.0(94.2-100)
p			0.40	0.19	0.11	0.046
Platelet count	$<20\times10^9$/L	35	2.8 (0.2-12.9)	18.7(7.2-34.4)	78.4(65.2-94.3)	90.5(80.7-100)
	$\geq20\times10^9$/L	74	4.1(1.1-10.4)	2.7(0.5-8.5)	93.2(87.7-99.1)	93.2(87.7-99.1)
p			0.76	0.01	0.044	0.78
Fetal hemoglobin (HbF)	<55%	82	3.6(1.0-9.4)	3.6(1.0-9.4)	92.7(87.2-98.5)	93.9(88.9-99.2)
	≥55%	20	5.0(0.3-21.1)	30.0(9.2-54.4)	65.0(44.8-94.2)	83.1(67.0-100)
p			0.78	0.001	0.004	0.19
Lung infiltration	Negative	30	3.3(0.2-14.8)	3.3(0.2-14.8)	93.3(84.8-100)	93.3(84.8-100)
	Positive	63	3.3(0.6-9.9)	11.6(5.0-21.3)	85.2 (76.6-94.7)	91.8(85.2-99.0)
p			0.96	0.23	0.32	0.88
Genetic mutation	Negative	2	0	0	100(100)	100
	PTPN11	63	1.6(0.1-7.6)	8.1(2.9-16.6)	90.4(83.3-98.0)	92.7(85.9-100)
	NF-1	20	10.0(1.6-27.8)	16.6(3.7-37.7)	73.3(55.6-96.8)	85.0(70.7-100)
	KRAS	13	7.6(0.4-30.3)	0	92.3(78.9-100)	92.3(78.9-100)
	NRAS	10	0	0	100	100
	CBL	1	0	0	100	100
p			0.53	0.59	0.33	0.73
Initial treatment regimen	DAC	46	6.5(1.7-16.2)	2.1 (0.2-10.1)	91.3(83.5-99.8)	93.4(86.5-100)
	C-DAC	63	1.5(0.1-7.6)	11.9(5.1-21.7)	86.6(78.2-95.8)	91.7(84.9-99.0)
p			0.17	0.09	0.58	0.85
Initial treatment clinical response	cCR/cPR	91	2.2(0.4-7.0)	2.3(0-5)	95.5(91.4-99.9)	96.2(92.1-1)
	cSD/cPD	18	11.1(1.7-30.4)	40.0(12.5-66.8)	48.9(27.6-86.7)	72.2(54.2-96.2)
P			0.064	<0.001	<0.001	<0.001
Bridging treatment clinical response	cCR/cPR	88	2.2(0.4-7.2)	2.3(0.4-7.4)	95.4(91.1-99.9)	96.0(91.5-100)
	cSD/cPD	18	11.1(1.7-30.4)	40.0(12.5-66.8)	48.9(27.6-86.7)	72.2(54.2-96.2)
p			0.07	<0.001	<0.001	<0.001
Molecular response (NGS VAF)	0.1%	6	0	0	100	100
	0.1-30%	33	0	3.0(0.2-13.6)	100	100
	>30%	24	4.2(0.3.4-18.0)	4.6(0.3-19.5)	91.3(80.4-100)	90.8(79.3-100)
p			0.44	0.87	0.55	0.29
RAS-related mutation gene number	0	2	0	0	100	100
	1	88	3.4(0.9-8.8)	5.7(2.1-12.0)	90.9(85.0-97.1)	91.5(85.5-97.8)
	2	17	5.8(0.3-24.2)	18.5(1.9-48.8)	75.6(53.4-100)	94.1(83.6-100)
	3	2	0	50(0.0-96.0)	50.0(12.5-100)	100
p			0.94	0.09	0.14	0.94
HSCT regimen	MDT	14	0	0	100	100
	CT	81	2.5(0.5-7.8)	9.1(3.9-16.9)	88.4(81.5-95.9)	93.8(88.7-99.2)
	LCT	14	14.3(2.1-37.5)	7.8(0.4-30.8)	77.9(58.7-1)	63.8(34.7-100)
p			0.07	0.57	0.20	0.051
Year of HSCT	Before2018	19	5.2(0.3-22.0)	21.1(6.3-41.6)	73.7(56.3-96.4)	84.2(69.3-100)
	After2018	90	3.3(0.9-8.6)	4.5(1.5-10.4)	92.1(86.7-97.9)	93.8(88.6- 99.3)
			0.69	0.02	0.02	0.14
KIR (centromeric)	A/A	25	/	8.0(1.3-22.9)	92.0(82.0-100)	96.0(88.6-100)
	B/X	76	/	8.7(3.4-17.1)	91.3(84.7-98.4)	95.6(90.7-100)
p				0.97	0.98	0.95
Thiotepa (TT)	Non-TT	89	3.4(0.9-8.7)	9.4(4.3-16.8)	87.3(85-94.6)	91.9(86.4-97.9)
	TT	20	5.0(0.3-21.1)	0	95.0(85.9-100)	95.0(85.9-100)
p			0.74	0.21	0.41	0.75
Engraftment	HLA-matched	14	/	0	100	100
	haploidentical	36	2.7(0.2-12.6)	13.9(4.9-27.3)	83.3(72.0-96.4)	88.9(79.2-99.8)
	UCB	58	3.4(0.6-10.7)	6.4(1.5-15.8)	90.3.6(82.4-99.0)	93.9(87.4-100)
p			0.78	0.14	0.21	0.34
Maintenance treatment (DAC)	<9 months	67	/	0	92.7(85.7-100)	96.0(90.5-100)
	≥9 months	34	/	7.3(2.2-16.8)	100	100
p				0.13	0.13	0.28
aGVHD	0-I	69	2.9(0.5-9.1)	6.9(2.1- 16.1)	90.1(82.6-98.3)	95.6(90.9-100)
	II	28	0	7.1(1.2-20.7)	92.9(83.8-100)	95.2(86.6-100)
	III-IV	12	16.6(2.3-42.7)	16.6(2.3-42.6)	66.7(44.7-99.5)	66.7(44.7- 99.5)
p			0.03	0.46	0.031	<0.001
cGVHD	Negative	71	1.4(0.1-6.8)	6.5(2.0-14.9)	92.1(85.5-99.2)	95.2(90.1-100)
	Limited	19	0	0	100	100
	Extensive	12	0	0	100	100
p			0.80	0.42	0.33	0.54
HSCT regimen	Engraftment					
CT	haploidentical	33	0	15.1(5.4-29.5)	84.8(73.5-98.0)	90.9(81.6-100)
	UCB	47	2.1(0.2-9.9)	5.3(0.9-15.9)	92.7(84.8-100)	97.9(93.8-100)
p			0.40	0.10	0.21	0.17

HSCT: hematopoietic stem cell transplantation; MDT: matched donor transplantation; CT: complementary transplantation; LCT: donor lymphocyte infusion bridging UCB transplantation; KIR: killer cell immunoglobulin-like receptor; UCB: umbilical cord blood; DAC: decitabine; aGVHD: acute graft-versus-host disease; cGVHD: chronic graft-versus-host disease

2 Supplementary Figures

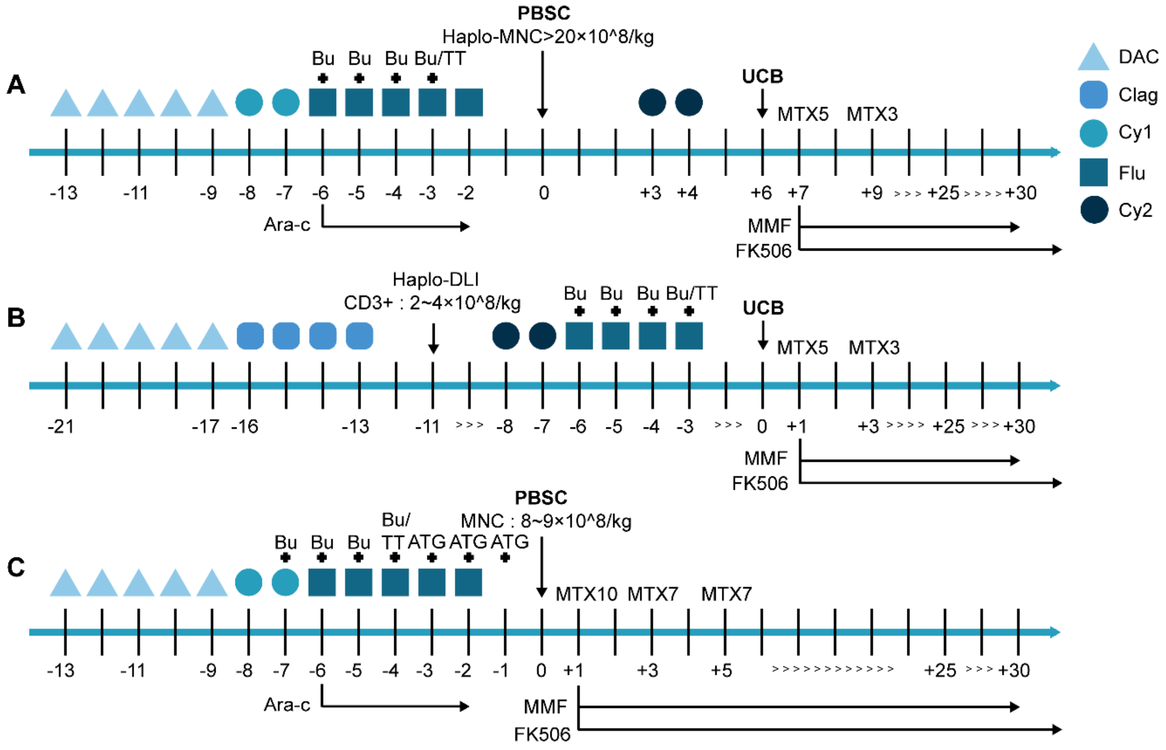

Supplemental Figure 1 Conditioning regimen and GVHD prophylaxis of three HSCT regimens

(A) Complementary transplantation (CT), (B) donor (haploid) lymphocyte infusion bridging UCB transplantation (LCT), and (C) matched donor transplantation (MDT).

DAC (decitabine): 20 mg/m^2/d, Clag: Cladribine 6~8 mg/m^2/d, Ara-C 1g/m^2/d, G-CSF 5μg/kg/d, Bu (busulfan): 100 mg/m^2/d (weight≤16 kg) or 3.6 mg/kg/d (weight>16 kg) targeting Bu-Css of 600~900 ng/mL, TT (thiotepa): 5 mg/kg bid, Cy1 (cyclophosphamide): 25 mg/kg/d, Cy2: 50 mg/kg/d, Flu (fludarabine): 30 mg/m^2/d (LCT), 40 mg/m^2/d (CT/MDT), ATG (Rabbit Anti-human T lymphocyte globulin): 15 mg/kg of total doses; Ara-C (cytarabine): 100 mg/m^2/d; MTX10/MTX7/MTX5/MTX3: 10/7/5/3 mg/m^2/d.

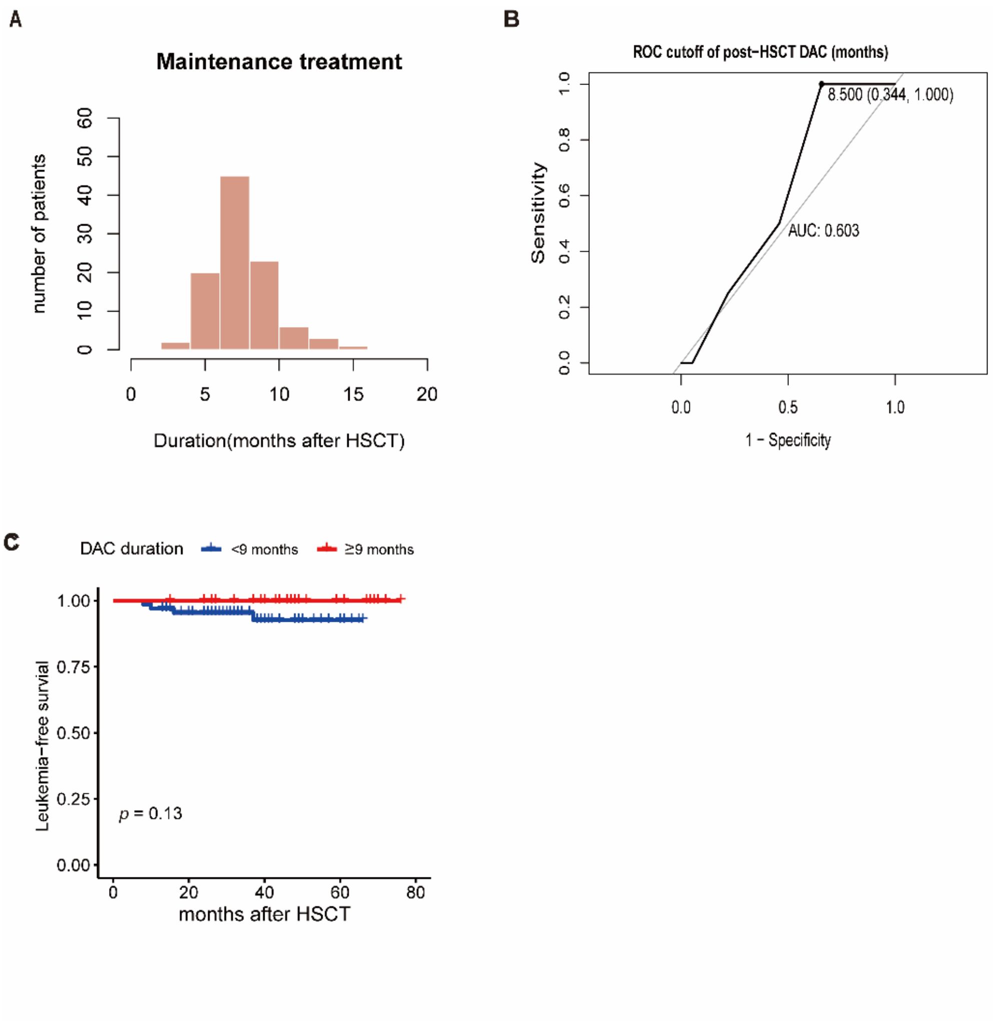

Supplemental Figure 2 Duration (months after HSCT) of low-dose DAC as post-HSCT maintenance treatment and survival of different duration

(A)Distribution of the 101 patients with different durations of DAC maintenance therapy, (B) Receiver Operating Characteristic (ROC) curve of DAC duration of LF: 8.5 months of cutoff value, (C) Comparison of LFS between DAC duration＜9months and ≥9months
